# Genetic and Epigenetic Diversity of *Pinus pinea* L.: Conservation Implications for Priority Populations in Greece

**DOI:** 10.3390/genes16040361

**Published:** 2025-03-21

**Authors:** Evangelia V. Avramidou, Ermioni Malliarou, Evangelia Korakaki, George Mantakas, Konstantinos Kaoukis

**Affiliations:** 1 Institute of Mediterranean Forest Ecosystems, ELGO-DIMITRA, Terma Alkmanos, Ilisia, 11528 Athens, Greece; ekorakaki@elgo.gr (E.K.); gmantakas@elgo.gr (G.M.); kkaoukis@elgo.gr (K.K.); 2 Forestry Research Institute of Thessaloniki, ELGO-DIMITRA, Vassilika, 57006 Thessaloniki, Greece; emalliarou@elgo.gr

**Keywords:** stone pine, MSAP, AFLP, Natura 2000 areas, conservation, population genetics and epigenetics

## Abstract

Background/Objectives: The stone pine (*Pinus pinea* L.) is an evergreen coniferous species valued for its edible seeds, which provide significant economic benefits to local populations. Remarkable phenotypic plasticity but low genetic variation characterizes the species. In Greece, natural populations of *P. pinea* are part of the Natura 2000 network and are protected under Annex I Priority Habitat type 2270. These populations, located across six Natura 2000 sites (including two islands), face increasing threats from tourism and climate change, leading to ecosystem degradation. Genetic and epigenetic studies are critical for the conservation of forest species because they provide insights into the genetic diversity, adaptive potential, and resilience of species, helping to inform effective management strategies and protect biodiversity in changing environments. This study aims to assess the genetic and epigenetic diversity of *P. pinea* in four Natura 2000 sites using molecular markers and to propose conservation strategies to ensure the species’ long-term sustainability. Additionally, a preliminary investigation of water potential under maximum daily water demand was conducted to evaluate the species’ adaptive response. Methods: Genetic analysis was performed using Amplified Fragment Length Polymorphism (AFLP) markers, while epigenetic analysis was conducted using Methylation-Susceptible Amplified Polymorphism (MSAP) markers. Sampling was carried out in four Natura 2000 areas, where genetic and epigenetic diversity patterns were examined. Furthermore, a preliminary study on water potential under peak daily water demand conditions was conducted to assess the species’ physiological adaptation to environmental stress. Results: The results of this study provide valuable insights into conservation strategies by highlighting the potential role of epigenetic variation in the adaptability of *P. pinea*, despite its low genetic variability. Understanding the species’ epigenetic flexibility can inform conservation efforts aimed at enhancing its resilience to environmental stressors, such as climate change. Additionally, the preliminary water potential analysis contributes to identifying physiological traits that may help predict the species’ survival under varying environmental conditions, guiding the development of more targeted conservation practices and management plans. Further research could refine these findings and strengthen their application in conservation efforts. Conclusions: The conclusions emphasize the critical importance of this research in informing conservation efforts for *P. pinea* in Greece, particularly considering climate change and human pressures. The results highlight the need for both in-situ and ex-situ conservation strategies to ensure the long-term sustainability of the species. The key recommendations include the protection of natural habitats, the implementation of controlled seed collection practices, and further research into the epigenetic mechanisms that may enhance the species’ resilience to environmental stress. Future studies should focus on deepening our understanding of these epigenetic factors and their role in the adaptability of *P. pinea*, which will be essential for developing more effective conservation measures.

## 1. Introduction

*Pinus pinea* L., commonly known as the stone pine, is a coniferous species distributed from Portugal’s Atlantic coast to the Black Sea and Mount Lebanon [1,2]. Its spread was historically influenced by human activity, particularly during the Roman era [3,4], and today, it occupies approximately 750,000 hectares across the Mediterranean basin [5]. It grows mainly in Spain, Turkey, Portugal, Italy, and to a lesser extent in Greece, Lebanon, Morocco, and France [6,7]. It grows at a wide range of altitudes, usually below 1000 m, but also occurs at higher altitudes, such as in Lebanon (1500 m) [2].

The stone pine (*P. pinea* L.) has been valued for centuries for its durable and wear-resistant wood, making it a preferred material for construction, furniture, and shipbuilding [8]. In addition to its timber, the species produces edible pine nuts, which are rich in protein, fat, ascorbic acid, thiamine, and riboflavin, providing significant nutritional and economic benefits to local communities [9,10]. Beyond timber and pine nuts, the stone pine yields other economically valuable products, such as resin and pinosylvin, which have industrial applications [11]. Its bark is utilized for tannin extraction, while pinecone shells and empty cones serve as biofuel through pyrolysis, offering a short-term economic alternative to timber harvesting [8,12]. Ecologically, the stone pine plays a crucial role in carbon sequestration, aiding climate change mitigation [13]. It also provides shade and habitat for various wildlife species. Adapted to arid conditions, its deep root system allows it to thrive in water-scarce environments and sandy soils, which promote root development better than heavy soils [2,14]. Additionally, the stone pine contributes to coastal ecosystem stability by preventing sand erosion, further highlighting its ecological significance [15]. Due to its aesthetic appeal and adaptability, it is widely cultivated as an ornamental tree in parks, streets, and gardens [16].

Genetics plays a crucial role in shaping pine characteristics, adaptability, and overall health. Genetic diversity, defined as the variety of genetic characteristics within a population, is essential for a species’ long-term survival and adaptability. In the stone pine, genetic diversity enables resilience against environmental challenges, such as disease, pests, and climate change. Greater genetic variation increases the likelihood that certain individuals possess traits crucial for survival and reproduction [4,8].

Despite its ecological and economic significance, *P. pinea* exhibits notably low genetic diversity, as reported in various studies. However, this limitation is counterbalanced by the species’ high phenotypic plasticity, particularly in response to heat and drought stress, which has facilitated its survival and expansion across diverse Mediterranean environments [2,17,18]. Effective conservation and management require a comprehensive understanding of the genetic structure and diversity of pine populations. The genetic studies of *P. pinea* began in 1995 with phage cloning, plasmid subcloning, and partial rDNA nucleotide sequencing [19]. This initial research provided a method for establishing phylogenetic relationships between *P. pinea* and closely related taxa. Krupkin et al. [20] classified Mediterranean species within the polyphyletic section *Pinea*, while Georgolopoulos et al. [21] identified a genetic marker (trnV-H/x-h) useful for the precise phylogenetic classification of 95 *Pinus* species, even in environments where DNA degrades. Other studies have employed principal component analysis (PCA) [22], isoenzymes [23,24], and microsatellites [25] to examine genetic variation within *P. pinea*. Vendramin et al. [26] suggested that a genetic bottleneck, followed by natural and human-facilitated dispersal, resulted in a widespread but genetically uniform species. Similar findings were reported by Pinzauti et al. [27]. A comprehensive review of the importance of *P. pinea* also is presented in [11].

While genetic research has provided valuable insights, it does not fully explain *P. pinea’s* adaptability. Recent studies have highlighted the role of epigenetics, particularly cytosine methylation, in plant response to environmental stress. Epigenetic modifications influence gene expression without altering DNA sequences, offering a complementary mechanism to traditional genetic adaptation. In conifers, cytosine methylation has been linked to developmental processes and environmental responses. Research on *P. pinea* revealed significant methylation levels, suggesting an important role in its adaptive potential [18]. Katsidi et al. (2023) further demonstrated that epigenetic diversity increased in response to pollution, highlighting its potential contribution to resilience under environmental pressures [28]. This study also highlighted the importance of methylation in the stone pine’s immediate epigenetic response when faced with pollution.

Understanding both genetic and epigenetic diversity is essential for conservation. Traditional genetic studies have been instrumental in identifying population structure and diversity, but they do not fully capture the mechanisms underlying adaptive potential. Epigenetic research adds another dimension by revealing how external factors influence gene expression, which is particularly relevant for species facing habitat degradation and climate change [29]. The integration of epigenetic insights into conservation strategies can enhance restoration efforts by identifying populations with greater adaptive potential based on their methylation patterns.

In Greece, natural populations of *P. pinea* are protected within the Natura 2000 network under Annex I Priority Habitat type 2270. These populations are distributed across six designated sites: (1) Skiathos Island (GR1430003), (2) Kolpos Lagana Zakinthou (GR2210002), (3) Limnothalassa Kalogrias, Dasos Strofilias (GR2320001), (4) Limni Kaiafa Kotixi (GR2330005), (5) Kiparissia (GR2550005), and (6) Ethniko Parko Schinia—Marathona (GR3000003). The conservation of *P. pinea* in these areas is crucial due to multiple environmental pressures, including habitat degradation, tourism impact, and biological invasions. The *P. pinea* populations in Greece are primarily located in sandy coastal regions, with two sites situated on islands. These habitats face significant anthropogenic pressures, particularly from tourism, which can lead to soil compaction, habitat fragmentation, and increased wildfire risks. Additionally, the invasion of *Pinus halepensis* poses a major ecological threat, as this species competes with *P. pinea* for resources, potentially altering forest composition and reducing regeneration success [30]. Another critical concern is the species’ low natural regeneration and limited seed production, which undermine its long-term viability. Effective conservation and restoration efforts are necessary to sustain these ecotypes. In response to these challenges, the Institute of Mediterranean Forest Ecosystems launched a restoration project in 2021, funded by the Schinias—Marathon National Park Management Agency, now part of the Natural Environment & Climate Change Agency (N.E.C.C.A.). The project tested different planting methods, including the use of 1-year-old and 7-year-old seedlings, plastic covering, hydrogel application, and fencing protection. However, the preliminary results indicated low seedling survival rates, underscoring the need for further enhancement measures to secure the species’ future in these habitats. Beyond direct human impacts, long-term environmental changes threaten *P. pinea* populations. A study by Gaitanis et al. [31] revealed that the forest cover in these regions has declined by over 47%, while wetlands have decreased by 37% in the past 60 years. Additional pressures stem from historical land-use changes, such as infrastructure developments for the 2004 Olympic Games and the construction of the Marathonian road. Furthermore, habitat degradation affects biodiversity, including local entomofauna, as highlighted by Petrakis et al. [32].

Given these conservation concerns, it is essential to investigate both the genetic and epigenetic diversity of *P. pinea* populations within the Natura 2000 sites. Genetic studies can help assess population structure, identify inbreeding risks, and inform seed sourcing for restoration. Epigenetic research is equally critical, as it provides insights into how *P. pinea* adapts to environmental stressors, such as climate change, pollution, and soil degradation. Understanding the interplay between genetic diversity and epigenetic modifications will allow for more targeted conservation strategies, ensuring the resilience and sustainability of *P. pinea* populations in Greece’s Natura 2000 network.

This study aims to bridge the research gap by integrating genetic and epigenetic analyses to assess the adaptability of *P. pinea* in Greek Natura 2000 sites. Specifically, we address the following key research questions: 1. What is the genetic diversity and population structure of *P. pinea* in these protected areas? 2. How does epigenetic variability contribute to the species’ adaptation to environmental stressors? 3. What conservation strategies can be proposed based on genetic and epigenetic insights to ensure the sustainability of *P. pinea* populations?

## 2. Materials and Methods

**Figure 1:**
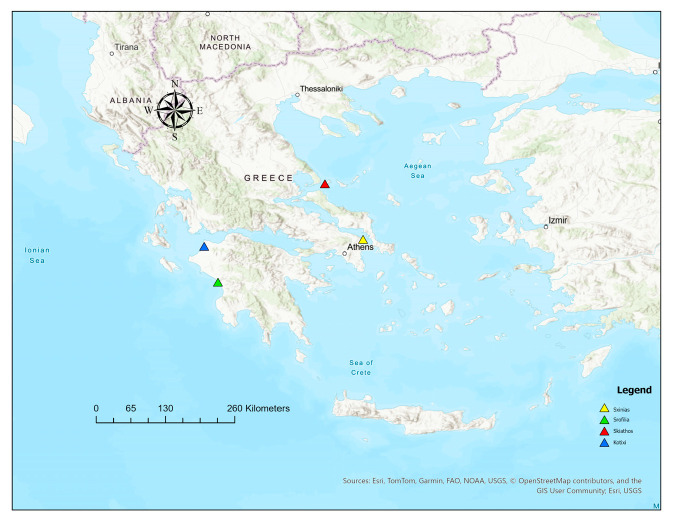
Presentation of the four *P. pinea* natural populations in the different areas of Schinias, Strofilia, Skiathos, and Kotixi.

**Figure 2:**
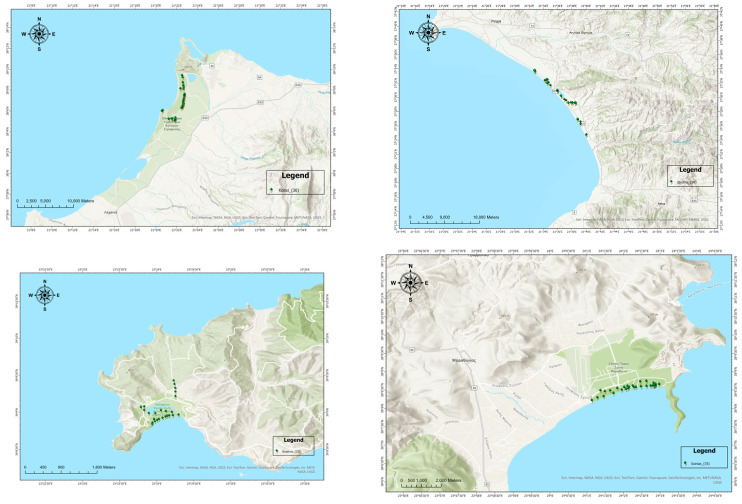
A detailed map showing *P. pinea* L. sampling genotypes across the four sites.

Four natural populations (Schinias, Skiathos, Kotixi, and Strofilia) were sampled to cover the major distribution of *P. pinea* (Figure 1 and Figure 2) in Greece. A total of 120 different trees were sampled (Schinias: 35; Skiathos: 25; Kotixi: 30; and Strofilia: 30). Needles were collected from trees at least 150 m apart and with vigorous phenotypes. The sites were chosen due to their ecological importance and the high disturbances they are facing due to anthropogenic pressures and the high touristic activities that threaten their sustainability in light of climatic changes. The *P. pinea* populations in Schinias, Skiathos, Kotixi, and Strofilia are ecologically significant as they support diverse ecosystems and protect the areas against soil erosion. In Schinias, a coastal area with wetlands, sand dunes, and forests provides habitat and food for various species. In Skiathos, the Strofylia Forest maintains biodiversity and safeguards soil. Kotixi, with its lagoons and marshes, houses one of the largest Pinus pinea populations, crucial for bird conservation. Strofilia, with the largest *P. pinea* forest in Greece, supports numerous species and maintains an ecological balance [33,34].

A plant DNA extraction kit (Macherey Nagel, Düren, Germany) was used to extract genomic DNA. The DNA concentration was determined using a UV spectrophotometer (Eppendorf Bio-Photometer, Hamburg, Germany). It was adjusted to a working concentration of 10 ng/μL. The DNA quality was also assessed through agarose gel electrophoresis to further examine if there was degradation, along with the values of 260/230 and 260/280 derived from the UV spectrophotometer. The sampled needles were immediately frozen to prevent changes in DNA methylation status. All samples followed the same protocol used for AFLP and MSAP marker analysis and underwent identical treatment for DNA extraction. Finally, we used negative controls to check for contamination during the amplification process. A sample without DNA was included to ensure that no amplification occurred in the absence of a template.

There is no meteorological station in Schinias, so the climatic conditions of the area are estimated using bioclimatic maps and data from the meteorological station in Rafina, located 12 km away. While using data from Rafina introduces potential inaccuracies, it is the closest station with comprehensive data, and its climatic conditions are considered reasonably representative of Schinias. According to the Rafina station data, the xerothermic period, defined as a warm and dry interval, lasts more than five months (April–September). In 2023, the annual rainfall was approximately 423.5 mm, with mean, minimum, and maximum temperatures of 19.2 °C, 6.1 °C, and 32.7 °C, respectively (data provided by the National Observatory of Athens;—https://meteosearch.meteo.gr/) (accessed on 1 June 2024). The maximum precipitation occurs in winter, followed by autumn, spring, and summer. The number of dry days ranges between 125 and 150, indicating that the bioclimate of the area is distinctly thermo–Mediterranean. The region belongs to the semi-arid bioclimatic zone with a mild winter (Q = 61.45 and m = 6.2), derived using the Worldwide Bioclimatic Classification System [35].

### 2.1. AFLP Procedure

The AFLP procedure consisted of three stages: (a) digestion of genomic DNA, (b) preamplification PCR, and (c) selective amplifications. For more details on the entire procedure, please refer to [36]. For information on the primers used in the current analysis, see Table 1. Moreover, three samples were used as replicate analyses, employing the same DNA extractions for AFLPs.

The f-AFLP product mixtures were denatured by heating in formamide at 94 °C for 2 min, followed by electrophoresis on an ABI Prism 3730xl Gene Analyzer (Applied Biosystems, Waltham, MA, USA). In total, ten selective AFLP combinations (Table 1) were used, with each genotype being scored to detect specific fragments. The size of the fragments was determined using the Genemapper v4.0 software and an internal size standard (GS 500 LIZ, Applied Biosystems, MA, USA). To reduce the influence of potential size homoplasy, only fragments in the range of 150 to 500 base pairs were included in the analysis [37].

### 2.2. MSAP Procedure

For the MSAP assay, we used EcoRI/HpaII or EcoRI/MspI restriction enzymes to double digest with the primers presented in Table 1. The whole procedure is described analytically in our previous publication [36]. Moreover, three samples were used as replicate analyses, employing the same DNA extractions for the MSAP analysis.

### 2.3. Needle Water Potential

Midday water potentials (Ψmd, the water potential during peak daily water demand) were recorded simultaneously for 13 trees out of 35 trees sampled from the Schinias natural population for genetic and epigenetic analysis. Two twigs per tree were collected, each with fully expanded, mature, sun-exposed leaves, and the measured Ψmd values were averaged per tree. The measurements were conducted between 12:00 and 14:00 using a portable pressure chamber (model PMS 1003, PMS Instruments, Corvallis, OR, USA), following the manufacturer’s guidelines.

### 2.4. Data Collection and Statistical Analysis

To convert allele size data from GeneMapper 4.0 (Applied Biosystems, USA) into a binary format, AFLP Excel macros were applied to mark allele sizes with 1 if present and 0 if not. To minimize the influence of potential size homoplasy, only reproducible fragments between 150 and 500 base pairs were considered and analyzed further [37].

For MSAP analysis, the comparison of banding patterns from the EcoRI/HpaII and EcoRI/MspI reaction results was presented in four possible fragment conditions, according to [38]. For the differentiation between the unmethylated and methylated fragments and for the evaluation of the specific effects of methylation conditions II and III, the mixed scoring 2 approach was used [38].

The R script MSAP_calc.r [38], with the default parameters listed on page 9 in the manual [39], was used to measure epigenetic diversity within populations. GenAlEx 6 [40] was utilized to calculate within-population haploid gene diversity (h). Additionally, GenAlEx facilitated the Analysis of Molecular Variance (AMOVA) for each subepilocus class, examining the variation in CCGG methylation states (epiloci) across eleven populations. Separate principal coordinate analyses (PCoAs) were conducted. To determine the relationship between matrices from the different marker systems, namely MSAPs and AFLPs, the standardized Mantel coefficient [41] was applied with 99 permutation tests to assess statistical significance. This test also measured the similarity between the geographic distance and the genetic distance, as well as the similarity between the geographic distance and the epigenetic distance [41].

To compare the mean values of Iepi and Ψmd across genotypes, regression analysis was conducted using needle water potential data with Sigmaplot (v.14.0, Systat Software Inc.). The Shapiro–Wilk test for normality was passed (*p* = 0.124), indicating that residuals follow a normal distribution.

## 3. Results

### 3.1. Genetic Diversity

For the four populations studied, eight AFLP selective primer combinations were used, yielding 880 fragments. The technical error rate from the replicate analysis was 2% for the AFLP replicate analysis. The mean percentage of polymorphism was 65.97% and ranged from 62.61% in the Strofilia population to 71.14% in the Schinias population, as shown in Table 2. The mean expected heterozygosity (He) ranged from 0.070 to 0.077 with a mean of 0.074. The Shannon diversity index (I) ranged from 0.131 (Kotixi population) to 0.143 (Schinias population). The number of effective alleles ranged from 1.093 (Kotixi population) to 1.104 (Schinias population), presenting a mean of 1.100.

**Figure 3:**
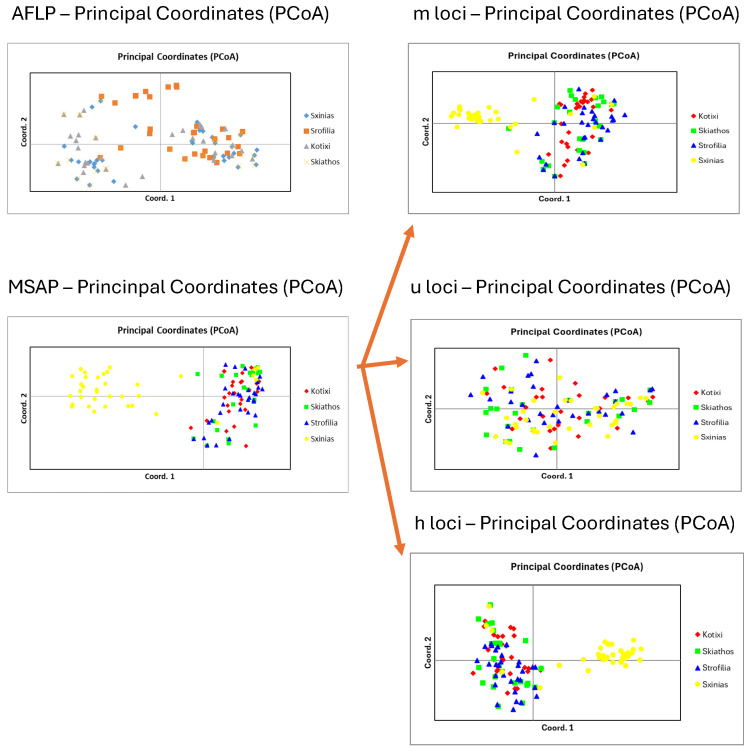
Principal coordinate analysis of genetics (AFLPs), epigenetics (MSAPs), and partition into three distinct methylation types: m-loci, u-loci, and h-loci.

The analysis of molecular variance revealed that 98% of the genetic variation occurred within populations, with only 2% found among populations (Table 3). Principal coordinate analysis accounted for 19.38% of the variance (Figure 3). The genetic diversity parameters, along with the epigenetic results, are presented in Table 2.

### 3.2. Epigenetic Diversity

Eight MSAP-selective primer combinations were used. The technical error rate from the replicate analysis was 2.2% for the MSAP analysis. They yielded 729 fragments for the four populations studied here. The number of markers per population ranged from 429 (Skiathos) to 564 (Schinias population). The mean percentage of the polymorphism was 64.51% and ranged from 58.71% for Skiathos to 77.37% for the Schinias population, respectively. The mean epigenetic expected heterozygosity H_epi_ ranged from 0.074 to 0.088 for Kotixi and Schinias, respectively. The epigenetic Shannon diversity index Iepi was 0.14 on average, ranging from 0.13 to 0.16 for the Kotixi and Schinias populations, respectively (Table 2).

Molecular variance analysis accounted for 91% of the epigenetic variation within populations and 9% only between populations (Table 3), whereas only 18.52% of the variance was explained by principal coordinate analysis (Figure 3).

The different parameters were calculated for each methylation profile, specifically for the u, m, and h alleles, using the mixed scoring 2 approach [39]. The details of each approach are shown in Table 4.

Separate principal coordinate analyses were carried out for the AFLP, MSAP, and various loci types—m, u, and h loci—and are illustrated in Figure 3. In the genetic analysis, the initial three axes accounted for 19.38% of the overall variation, indicating a lack of population differentiation. Meanwhile, in the epigenetic analysis, the first three axes explained 18.52% of the total variation, with m, u, and h loci individually contributing 27.52%, 26.21%, and 20.34%, respectively.

### 3.3. Correlation Between Geographic—Genetic and Epigenetic Variability

**Figure 4:**
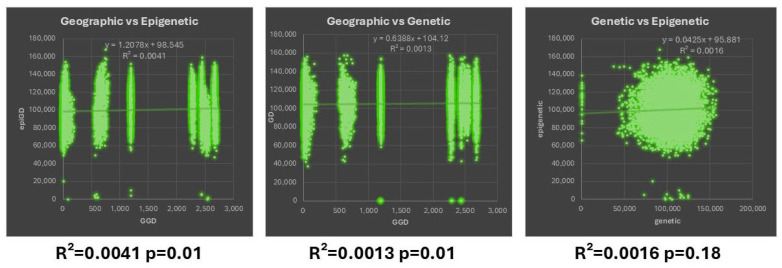
Mantel test between geographic, genetic, and epigenetic distances.

The Mantel test was used for the correlation between the genetic and epigenetic variability (Figure 4). There was a non-significant positive correlation (R^2^= 0.041, *p* = 0.01) between the pairwise epigenetic (MSAPs) and geographic distances, indicating that the observed positive correlation could have occurred by chance, so it does not provide strong evidence of a meaningful relationship between the two variables. Similarly, a non-significant positive correlation (R^2^ = 0.0013, *p* = 0.01) between the genetic (AFLPs) and geographic distances was found. Lastly, there was no correlation between the genetic and epigenetic distance matrices assessed from the AFLPs and MSAPs, indicating that almost none of the variation in one variable is explained by the variation in the other (R^2^ = 0.0016, *p* = 0.18).

### 3.4. Correlation Between Epigenetic Variability and Tree Water Status

**Figure 5:**
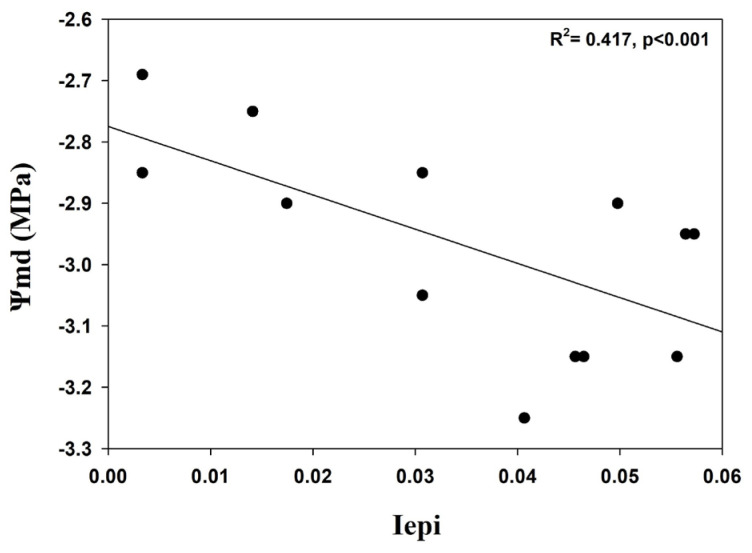
Correlation between epigenetic Shannon diversity index (Iepi) and needle water potential (Ψmd).

Regression analysis was used to test the correlation between Iepi and Ψmd by comparing the mean values of thirteen stone pines in the Schinias population. A significant negative correlation was observed (R^2^ = 0.417, *p* < 0.001; Figure 5). In contrast, plotting Ψmd against the genetic diversity indices revealed non-significant relationships, suggesting that the species’ genetic diversity was not influenced by variations in tree water status.

## 4. Discussion

Biodiversity, evolution, and adaptation are all based on genetic diversity. Many recent studies indicate that epigenetic regulation plays an important role in adaptation [42,43,44] and phenotypic plasticity. Complicating efforts to elucidate the role of DNA methylation is the fact that variation in DNA methylation may or may not be dependent on the underlying genetic variation in the DNA sequence [18]. According to Richards [42], there are two main extreme relationships between genetic and epigenetic variation that have profound implications for adaptation. Under climate change, an in-depth analysis of genetic and epigenetic diversity and its interrelationships will be critical to forest resilience and adaptation [45,46,47]. The conservation and protection of natural resources should incorporate genetic and epigenetic studies to initially define forest priority populations [45,46,47,48,49]. Recently, Avramidou et al. [36] also incorporated genetic and epigenetic analysis in order to propose the first planning conservation actions, both in situ and ex situ, for *Juniperus drupacea*, which is labeled as endangered according to the IUCN in Europe. Furthermore, environmental conditions may play an important role in facilitating adaptive evolution, as shown in another study on *Betula ermanii* [50], where populations from two contrasting habitats showed significantly different genetic and epigenetic population structures. So, studying genetics and epigenetics can also help define the adaptation mechanisms of forest tree species [43,51] and also define the mechanisms of longevity [52].

Previous research has consistently reported low genetic variation in *P. pinea* [7,26,53]. Our findings corroborate with this, showing limited genetic diversity across populations, as observed in the Schinias population (He = 0.077). This aligns with earlier studies that used AFLP markers and detected no polymorphic markers, reinforcing the species’ low genetic diversity [18]. However, the epigenetic diversity was notably higher (Hepi = 0.088), suggesting that epigenetic mechanisms may play a crucial role in *P. pinea’s* adaptability.

Interestingly, our methylation-sensitive amplified polymorphism (MSAP) analysis revealed that approximately 65% of cytosines at CCGG motifs were methylated. This high level of cytosine methylation has been proposed as an alternative mechanism for phenotypic plasticity in species with low genetic diversity [18]. The lack of correlation between genetic and epigenetic diversity in our study is consistent with the hypothesis proposed by [42], which suggests that epigenetic variation can be independent of genetic variation but significantly influences plasticity.

Studying the correlation between genetic and epigenetic factors is crucial for understanding gene regulation, environmental impacts, disease mechanisms, and evolutionary processes. This integrated approach reveals how genes are regulated, how organisms respond to environmental changes, how diseases develop, and how species adapt. Herein, we observed no significant correlation between the genetic and epigenetic distances, which is also in accordance with the theory of Rirchards [42], who stated that genetic diversity can be completely uncoupled from epigenetic diversity but probably has a larger effect on the plasticity of the species. The most important finding was that the genetic variability was completely uncoupled from the epigenetics in this species, which also confirms the findings of Sáez-Laguna et al. [18]. This result indicates the potentially important role of epigenetic variability as an evolutionary mechanism for the species.

Moreover, according to our results on genetic and epigenetic diversity, the Schinias population had higher values of genetic (He = 0.077) and epigenetic (Hepi = 0.088) parameters, probably indicating that the first establishment of the stone pine in Greece initiated from that area, which was also a significant harbor in ancient times and a famous battlefield (590 BC). Nevertheless, the genetic diversity of all the populations also presented low values, in agreement with previous research [18] on the species, and did not group the populations according to principal coordinate analysis (Figure 3). In anticipating the methylation analysis for all the loci, the m and h loci formed a group in the Schinias population (Figure 2).

Furthermore, the low genetic diversity of the stone pine likely suggests a stronger environmental influence on phenotypic traits, contributing to the species’ adaptability. This environmental effect, which may be mediated by epigenetic changes, is consistent with the findings of Mutke et al. [54], who studied experimental sites established over a decade ago in 40 Lebanese, Turkish, Greek, Italian, Spanish, Portuguese, Moroccan, and Tunisian locations. Their research showed that when assessing height as a growth parameter, the samples were uniform among the provenances but displayed significant differences among the sites. These results indicate that the stone pine’s growth is influenced more by environmental conditions like soil conditions and site characteristics than by genetic differences among populations.

Additionally, a preliminary analysis of midday water potentials (Ψmd, water potential under maximum daily water demand) was performed in this study to examine water potential correlation with genetic and epigenetic parameters. We found a significant negative correlation between the epigenetic Shannon index and Ψmd, but no significant correlation was found for the genetic Shannon index, indicating that the genetic diversity of the species was not influenced by different tree water status. The negative correlation between the epigenetic Shannon index and Ψmd may be the result of the species adapting to drought, also pointed out by similar studies. For example, Do et al. [55] found that methylated 5-hmC and a ratio of 5-hmC/5-mC were significantly negatively correlated with changes in water potential in *P. radiata*, and a significant negative correlation was observed between stomatal conductance and 5-mC. Furthermore, according to Simões et al. [11], the stone pine has a high tolerance to drought and can be considered a model tree for climate change adaptation and reforestation efforts. A number of different studies, also in trees [56,57], such as poplar [56], apples [58,59], etc., pinpoint the significant relation between water deficit and epigenetic changes. Further studies concerning the water needle potential and epigenetic parameters should be performed to investigate the detailed relationship between them.

Despite our findings, several methodological limitations must be acknowledged. First, our sampling strategy may introduce biases, as it may not fully capture the entire genetic and epigenetic variability in *P. pinea* across its range, but only to four from the six Natura 2000 areas. Future studies should employ broader geographic sampling to improve all the protected areas. Second, the choice of markers (AFLPs and MSAPs) has some limitations beyond their dominant character. Specifically, MSAP provides only a partial view of epigenetic modifications, as it focuses exclusively on DNA methylation at specific restriction sites [60]. Future research should integrate whole-genome bisulfite sequencing to obtain a more comprehensive epigenetic view of the genome. Furthermore, while we observed no correlation between genetic and epigenetic diversity, a deeper mechanistic understanding is required. Functional genomic studies exploring how epigenetic modifications influence gene expression and adaptive traits will be crucial in confirming the role of DNA methylation in *P. pinea’s* resilience. Additionally, long-term monitoring of epigenetic changes in response to environmental stressors, such as drought, could provide valuable insights into the stability and heritability of these modifications [45].

Although ecotype 2270 of the species is protected as an Annex I site in the European Natura 2000 network, further measures are needed to ensure the viability of the species in the face of climate change and human pressures. Our recommendation is a two-fold strategy, which depends on our current genetic and epigenetic results for the species. We propose establishing in situ and ex situ protected areas for the Natura site GR3000003 Ethniko Parko Schinia—Marathona. The areas should be in situ (by establishing at least two subpopulations of priority in the area) and ex situ (collection and preservation of seeds) protected due to their highest genetic and epigenetic diversity in comparison to other Natura areas. Moreover, a selection of superior genotypes by grafting should be planted in another area with similar climatic conditions to back up the genetic pool of the area. Additionally, the genotypes situated on Skiathos Island (GR1430003) should also be protected ex situ through seed collection, since the touristic pressure that the area faces is significantly higher every year. Moreover, for the two other Natura areas, Dasos Strofilias, GR2320001, and Limni Kaiafa Kotixi, GR2330005, we also propose ex situ conservation of the seeds. Finally, for the other two unstudied areas, we should proceed with further genetic and epigenetic analysis to have a complete view of the species in the priority Natura 2000 areas for ecotype 2270.

## 5. Conclusions

Genetic diversity has long been regarded as the main contributor to the biodiversity and adaptive capacity of ecosystems. However, recent studies have highlighted the role of epigenetics in adaptation and phenotypic plasticity, complicating the understanding of how genetic and epigenetic factors interact. Variations in DNA methylation can occur independently of genetic differences, making it essential to analyze both genetic and epigenetic diversity to better understand how forest ecosystems can adapt to climate change. The stone pine (*P. pinea* L.) is characterized by low genetic variation and high phenotypic plasticity, a combination that raises interesting questions about its evolutionary strategies. Extensive studies have shown negligible genetic diversity, particularly in chloroplast microsatellites, but significant levels of DNA methylation have been recorded, indicating that epigenetic variability may correlate with adaptive traits. In this study, we examined four Natura 2000 priority areas with stone pines, ecotype 2270. Our findings were that the Schinias population has the highest level of genetic and epigenetic diversity, without significant correlations between the two diversities, supporting the theory that genetic and epigenetic diversity are uncoupled.

Our results suggest that low genetic diversity in stone pine populations may enhance their adaptability to environmental conditions, likely influenced by epigenetic factors. Preliminary analyses indicate a negative correlation between epigenetic diversity and midday water potential, highlighting the species’ ability to adapt to drought. Given the low genetic diversity of the stone pine, conservation strategies should focus on increasing the species’ resilience to climate change through the establishment of both in situ and ex situ conservation areas, including the creation of subpopulations and seed preservation in Ethniko Parko Schinia-Marathonas and other Natura areas. These findings support the hypothesis that genetic and epigenetic diversity are uncoupled and underscore the importance of incorporating epigenetic analyses in conservation efforts to better understand and enhance the species’ adaptability.

## Figures and Tables

**Table 1:** The EcoRI/MseI and HpaII/MspI adapters, as well as the pre-selective and selective primers used for the AFLP and MSAP analysis.

Primer Name	5′ to 3′ Sequence
EcoRI adapter	CTCGTAGACTGCGTACC AATTGGTACGCAGTC
MseI adapter	GACGATGAGTCCTGAG TACTCAGGACTCAT
HpaII/MspI adapter	GACGATGAGTCTCGAT CGATCGAGACTCAT
Pre-selective EcoRI primer	GACTGCGTACCAATTC-A
Pre-selective MseI primer	GATGAGTCCTGAGTAA-C
Pre-selective HpaII/MspI primer	ATGAGTCTCGATCGG-A
Selective EcoRI primers	GACTGCGTACCAATTC + ATG (FAM) GACTGCGTACCAATTC + ACT (HEX) GACTGCGTACCAATTC + AAC (ROX) GACTGCGTACCAATTC + AAG (TAMRA)
Selective MseI primer	GATGAGTCCTGAGTAA-CAA GATGAGTCCTGAGTAA-CAC GATGAGTCCTGAGTAA-CGT GATGAGTCCTGAGTAA-CTC
Selective HpaII/MspI primer	ATGAGTCTCGATCGGATC ATGAGTCTCGATCGGACT ATGAGTCTCGATCGGAAT
EcoRI adapter	CTCGTAGACTGCGTACC AATTGGTACGCAGTC

**Table 2:** Collection sites of *P. pinea* populations, total epigenetic diversity, and a comparison with genetic diversity indices in the same individual plants (P_epi_: the percentage of polymorphic subepiloci, I_epi_: Shannon’s information index based on the epiloci, He and H_epi_: haploid genetic and epigenetic diversity, P: the percentage of polymorphic bands, I: Shannon’s diversity index based on the genetic and epigenetic loci, N.B.: the number of bands, and N.P.B.: the number of private bands).

	AFLP			MSAP				
Population	P	I	He	P_epi_	I_epi_	H_epi_	N.B.	N.P.B.
Schinias	71.14	0.14	0.077	77.37	0.16	0.088	564	122
Strofilia	62.61	0.14	0.074	61.73	0.14	0.080	452	29
Kotixi	64.09	0.13	0.070	60.22	0.13	0.074	441	34
Skiathos	66.02	0.14	0.076	58.71	0.14	0.078	429	13
Mean	65.97	0.14	0.074	64.51	0.14	0.080	471.5	49.5

**Table 3:** Hierarchical AMOVA for AFLP and MSAP data (all subepiloci, as well as different subepiloci classes separately) performed by grouping populations according to regions of origin.

Loci/Groups	Source of Variation	d.f.	Variance Component	Total Variance (%)	Φ-Statistics (Φ_ST_)	*p*-Value
**AFLP loci**	Among Populations	3	0.835	2	0.016	>0.001
	Within Populations	116	52.891	98		
	Total	119	53.726	100		
**MSAP all subepiloci**	Among Populations	3	4.862	9	0.095	>0.001
	Within Populations	116	46.514	91		
	Total	119	51.376	100		
**MSAP *m*-subepiloci**	Among Populations	3	1.386	13	0.129	>0.001
	Within Populations	116	9.390	87		
	Total	119	10.776	100		
**MSAP *h*-subepiloci**	Among Populations	3	2.540	11	0.114	>0.001
	Within Populations	116	19.677	89		
	Total	119	22.217	100		
**MSAP *n*-subepiloci**	Among Populations	3	0.000	0	0.000	<0.001
	Within Populations	116	7.579	100		
	Total	119	7.579	100		

**Table 4:** Different methylation alleles: u, m, and h alleles; Ne: number of effective epigenetic alleles; I_epi_: epigenetic Shannon’s diversity index, and H_epi_: haploid epigenetic diversity.

	U Alleles			M Alleles			H Alleles		
Population	Ne	I	He	Ne	I	He	Ne	I	He
Kotixi	1.174	0.178	0.110	1.139	0.167	0.096	1.097	0.134	0.072
Skiathos	1.186	0.193	0.118	1.155	0.190	0.109	1.098	0.129	0.071
Strofilia	1.185	0.202	0.122	1.182	0.206	0.122	1.094	0.127	0.069
Schinias	1.168	0.196	0.114	1.131	0.193	0.102	1.106	0.155	0.082
Mean	1.178	0.192	0.116	1.152	0.189	0.107	1.099	0.136	0.074

## Data Availability

The original contributions presented in the study are included in the article, further inquiries can be directed to the corresponding author.
